# Estrategias de fortalecimiento de la seguridad y la soberanía alimentaria en medio de la pandemia de COVID-19 en Colombia

**DOI:** 10.7705/biomedica.6041

**Published:** 2022-05-01

**Authors:** Paula Andrea Castro, Julie Pauline Bustos, Paola Rueda-Guevara

**Affiliations:** 1 Centre d’Estudis Demográfics, Universitat Autónoma de Barcelona, Bellaterra, España Universitat Autónoma de Barcelona Universitat Autónoma de Barcelona Bellaterra Spain; 2 Salud Poblacional, Fundación Santa Fe de Bogotá, Bogotá, D.C., Colombia Fundación Santa Fe de Bogotá Bogotá, D.C. Colombia; 3 Consultora independiente, Bogotá, D.C., Colombia Consultora independiente Bogotá, D.C. Colombia

**Keywords:** seguridad alimentaria y nutricional, abastecimiento de alimentos, pandemias, Food and nutrition security, food supply, pandemics

## Abstract

En medio de la emergencia sanitaria producida por la COVID-19 en Colombia, se generaron estrategias para el fortalecimiento de la seguridad y la soberanía alimentaria. Entre los mecanismos adoptados por las comunidades urbanas, rurales y los gobiernos locales se encuentran las formas alternativas en la obtención de alimentos -como el trueque- el cual ha facilitado el intercambio entre territorios de alimentos producidos por los campesinos. A su vez, en Bogotá, se han estado fortaleciendo los mercados campesinos que han permitido llevar a los hogares urbanos alimentos producidos por los campesinos de Tolima, Meta y Boyacá fomentando los circuitos cortos de comercialización y el pago justo a los productores. A pesar de estas alternativas, las manifestaciones para visibilizar el hambre de los hogares se han hecho presentes. Un ejemplo de ello fueron los trapos rojos en las puertas y ventanas de las casas que alertaron sobre la situación de buena parte de colombianos y han alentado la solidaridad entre vecinos.

Finalmente, la posición de la academia ha sido vanguardista vinculando de manera directa a los campesinos en procesos por la garantía del derecho a la alimentación en Colombia.

## Abstrac

In the midst of the health emergency caused by COVID-19 in Colombia, strategies have been generated to strengthen food security and sovereignty. Among the mechanisms adopted by urban and rural communities and local governments are alternative ways of obtaining food, one of which is trueque, which allows the exchange of food produced by peasants between territories. In Bogotá, the mercados campesinos have been strengthened, which have made it possible to bring food produced by peasants in Tolima, Meta and Boyacá to urban households, promoting short marketing circuits and fair payment to producers. In spite of these alternatives, the demonstrations to make visible the hunger of the households emerged the trapos rojos (red rags) in the houses, which have alerted about the situation of a good part of Colombians and have encouraged solidarity among neighbors.

The position of academia has been avant-garde, directly involving peasants in processes to guarantee the right to food in Colombia.

Colombia, como muchos otros países de la región y del mundo, decretó el aislamiento preventivo obligatorio el 24 de marzo del 2020 ^1^ para hacer frente a la velocidad de contagio de la COVID-19 y, durante más de un año, el país se aisló de manera intermitente para frenar su contagio ^2^. Por cuenta de este confinamiento, en los hogares colombianos se incrementó la inseguridad alimentaria, ya vergonzosa desde antes de la pandemia, pues su prevalencia era del 52 % a nivel nacional según datos de la Encuesta de Situación Nutricional -ENSIN- 2015^3^.

En la actualidad, la inseguridad alimentaria sigue siendo una constante para las personas en condiciones de vulnerabilidad. En febrero del 2021, el Departamento Administrativo Nacional de Estadística (DANE) reveló que tan solo el 2 % de los hogares vulnerables en Colombia cubrían sus necesidades alimentarias, pues 2,4 millones de personas ingerían menos de tres comidas diarias con respecto a marzo del 2020, cuando se inició la pandemia. Además, cerca de 9.000 hogares no tendrían siquiera cómo cubrir una comida diaria ^4^. Es pertinente anotar que las condiciones socioeconómicas de muchos de los hogares son precarias por los niveles de informalidad laboral y desempleo, que según los datos disponibles, se ubicaban en el 49,2 y el 15,9 %, respectivamente, en febrero del 2021, es decir, un aumento de cerca de un punto porcentual en la primera y de tres en el segundo, con respecto al año inmediatamente anterior ^5^.

En respuesta a esta crisis, el aparato institucional desarrolló estrategias como la entrega de paquetes alimentarios para las familias, especialmente las más vulnerables. En Bogotá, la Secretaría de Integración Social entregó paquetes alimentarios de contingencia para 26 días, con un aporte de 1.080 calorías diarias ^6^. En Medellín, la entrega de paquetes alimentarios se canalizó a través del Programa de Buen Comienzo, enfocado, en este caso, en niños menores de cinco años, mujeres gestantes y madres lactantes ^7^, en tanto que instituciones privadas como la Fundación Éxito entregaron más de 48.000 paquetes de alimentos para la primera infancia vulnerable ^8^. También, se fortalecieron ejercicios de organización social que han originado estrategias para garantizar el derecho a la alimentación.

Cabe resaltar que las secretarías de atención social de ciudades y municipios entregaron apoyos alimentarios con base en estrategias diseñadas desde antes de la pandemia para facilitar el acceso y el consumo de una alimentación completa, equilibrada, suficiente, adecuada e inocua, por lo que no constituyeron un mecanismo novedoso. Por el contrario, antes de la pandemia, los bonos monetarios canjeables por alimentos incluían: los paquetes con aceite, arroz y leguminosas, los comedores comunitarios y la entrega de comida caliente eran programas existentes, por ejemplo, en Bogotá ^9^. Dichas medidas, sin embargo, deben complementarse con otras que potencien la autonomía y la soberanía alimentaria, y reconozcan los contextos locales de un país tan diverso como el nuestro, en aras de garantizar el derecho a la alimentación.

La seguridad alimentaria y nutricional es la disponibilidad, el acceso y el consumo oportuno, permanente, suficiente, estable e inocuo de alimentos, en la cantidad y calidad necesarias para su adecuada utilización biológica y para llevar una vida saludable y activa ^10^. Además, según Nyélény, la soberanía alimentaria es:

“El derecho de los pueblos a alimentos nutritivos y culturalmente adecuados, accesibles, producidos de forma sostenible y ecológica, y su derecho a decidir su propio sistema alimentario y productivo. Esto pone a aquellos que producen, distribuyen y consumen alimentos en el corazón de los sistemas y políticas alimentarlas, por encima de las exigencias de los mercados y de las empresas. Defiende los intereses de, e incluye a, las futuras generaciones” ^11^.

Estos dos enfoques se complementan y dan cuenta de lo que sería el goce efectivo del derecho a la alimentación.

En este contexto, el objetivo del presente ensayo fue describir ejemplos de liderazgo comunitario que han contribuido a garantizar el derecho a la alimentación y la seguridad y soberanía alimentaria, y fortalecer el tejido social en Colombia en el marco de la emergencia sanitaria de la COVID-19.

## Formas alternativas en la obtención de alimentos

La forma de obtener los alimentos ha sido el cambio más importante en el sistema agroalimentario colombiano de cara a la emergencia por la COVID-19. Algunos mecanismos adoptados por las comunidades urbanas y rurales, así como por los gobiernos locales, han resultado muy acertados para abastecer a la población. Entre ellos, se destaca el trueque y el uso de las redes sociales y plataformas que acercan a los productores y los consumidores.

Es el caso de los mercados Itinerantes que no requieren de mobiliario fijo, los mercados campesinos permanentes en las tradicionales plazas de mercado y los mercados campesinos alternativos que recurren a plataformas digitales o al servicio a domicilio ^12^.

Asimismo, vale la pena mencionar la donación de alimentos a los bancos de alimentos y las formas alternativas que contribuyen a la prevención y reducción de pérdidas y desperdicios de alimentos, tales como los protocolos de asesoría técnica y gestión del crédito para empresas, el acompañamiento a las pequeñas superficies y tiendas de barrio, y la educación para evitar el desperdicio de alimentos, todas estas iniciativas lideradas por las entidades gubernamentales ^13^.

Sin embargo, también se han observado desaciertos institucionales, pues en pos de satisfacer las necesidades nutricionales de las comunidades, se ha favorecido a la industria alimentaria por encima de los productores locales, como se evidencia en la asistencia alimentaria brindada por el gobierno de Bogotá en unión con una industria alimentaria en julio del 2020 ^14^.

## Trueque

Con la aparición de la agricultura en el neolítico, se introdujo el trueque como una estrategia de intercambio alimentario que permitía compartir alimentos y saciar el hambre, además de expandir costumbres y construir territorio ^15^. La recuperación del trueque es positiva en términos alimentarios, aunque no elimine la necesidad del dinero. Desde el inicio de la pandemia se ha detectado la práctica del trueque en algunos municipios del departamento de Cundinamarca: en los municipios de Ubaté y Útica, se intercambiaron 1.800 panelas por 4.000 yogures; en Villapinzón y Villeta, se cambiaron 105 bultos de papa por 50 cajas de panela; en Sopó, un grupo de mujeres confeccionó tapabocas que fueron entregados en Guasca a cambio de 12 bultos de zanahoria, papa y mazorca. Estos trueques han sido impulsados por los gobiernos locales, por ejemplo, durante el 2020 y parte del 2021, Jaime Torres, alcalde de Ubaté, los promovió con los municipios de Bituima y Mesitas del Colegio, entre otros ^16^. En Frontino (Antioquia), el trueque comenzó a hacerse dentro del municipio cuando un voluntario colocó dos mesas frente a su casa, una con alimentos para quien los requiriera y otra para la donación voluntaria de alimentos ^17^.

En ese sentido, el Consejo Regional Indígena del Cauca ha planteado cómo los trueques y los intercambios en la comunidad han permitido hacer frente a la crisis ^18^. Al norte del país, en la Sierra Nevada de Santa Marta, los indígenas kankuamos empezaron a intercambiar productos ancestrales como mochilas de fique y lana por alimentos no perecederos y útiles básicos de aseo a un precio justo, en lo que constituye no solo un ejercicio de trueque sino de recuperación y preservación de saberes ancestrales ^19^.

## Mercados campesinos

Otra estrategia importante ha sido el fortalecimiento de los mercados campesinos. En Bogotá, ante el aislamiento decretado como medida de control del Estado contra la propagación del coronavirus, surgieron dinámicas de reorganización de los mercados campesinos mediante las cuales las asociaciones campesinas organizaron, conjuntamente con la Alcaldía Mayor y la Secretaría de Desarrollo Económico, mercados campesinos móviles.

Esta iniciativa transformó el mercado campesino tradicional, basado en la instalación de pequeños puestos para la venta de productos en carpas que se levantan temporalmente en espacios de la ciudad, con el fin de distribuir los productos de la canasta básica familiar por medio del servicio a domicilio: el comprador solicitaba por teléfono o por internet los alimentos, el pedido era enviado a su hogar y allí se efectuaba el pago. Por este medio se comercializaron más de 660 mercados en 14 localidades de la capital ^20^, con lo que se contribuyó a la seguridad alimentaria de las familias, se redujo la cadena de comercialización evitando los intermediarios, y se benefició directamente a los campesinos ^21^, pues los mercados provenían de los departamentos de Boyacá, Tolima y Meta.

Para cumplir con la estrategia de los mercados campesinos itinerantes, la gobernación de Boyacá compró 7.000 mercados directamente a los campesinos productores sin que ellos tuvieran que desplazarse, asumiendo así el rol de intermediario, para evitar que el valor de los alimentos se perdiera para ellos en la cadena de venta ^22^, y contribuyendo a aliviar las dificultades económicas que los campesinos sufrían desde antes de la cuarentena ^23^.

## Trapos rojos

Los trapos rojos que la comunidad colgaba de las puertas y ventanas de las casas durante la pandemia en los barrios periféricos y marginados de Bogotá y varias partes del país, sirvieron para revelar el hambre de los hogares. Ello permitió que las personas se manifestaran y que la ciudadanía fortaleciera indirectamente una posición política frente a la carencia de alimentos y bienes básicos ^24^. Además, condujo a un ejercicio de solidaridad entre vecinos que fortalece el tejido social. En Paipa (Boyacá), en el 2020 se utilizaron los trapos rojos para visibilizar el hambre y los trapos verdes para identificar a quienes podían y deseaban donar alimentos. Fue un gran número de hogares los que estuvieron dispuestos a brindar alimentos a los vecinos que lo requerían ^25^.

Los trapos rojos han aparecido en varios de los municipios que el DANE clasifica como los de mayor inseguridad alimentaria, específicamente en ciudades costeras como Sincelejo, Santa Marta, Cartagena, Montería y Barranquea, además de Bogotá. En estos sitios, más del 90 % de la población consumía tres comidas diarias antes de la pandemia y hoy ese porcentaje ha disminuido entre 20 y 50 puntos ^4^, lo que estaría relacionado con el aumento del desempleo y la disminución de ingresos en los hogares y, por ende, de su capacidad para la compra de alimentos.

## Posición de la academia

La academia ha propuesto mecanismos para garantizar la seguridad alimentaria durante la pandemia. Por ejemplo, el Observatorio Rural de la Universidad de La Salle sostiene que es necesario reconocer al campesinado como sujeto político y social, propiciando su capacidad y su identidad cultural asociada con la producción de alimentos, y reconociendo su papel para garantizar la alimentación en el país ^26^. Es necesario empezar a implementar estrategias de agricultura urbana, ya que, además de la producción de alimentos, se fortalece el tejido social con la integración de personas sin conocimientos previos o con conocimientos en otros campos ^27^.

## Conclusiones

Las estrategias generadas espontáneamente o por influencia institucional para incentivar formas alternativas de obtención de alimentos y fortalecer los circuitos de comercialización que vinculan el campesinado, sin duda, han sido herramientas indispensables para limitar el aumento de la inseguridad alimentaria en el país a causa de la COVID-19. De cara a futuras pandemias, estas herramientas servirían para fortalecer el aparato institucional y político alrededor del derecho a la alimentación en Colombia.

Cabe resaltar que el trueque es una práctica aborigen que aún se preserva en comunidades indígenas como los kokonucos en el Cauca, quienes consideran que el trueque constituye una forma de hacer frente a los tratados de libre comercio, y fortalecer la soberanía y autonomía alimentaria ^15^. En este sentido, en las comunidades campesinas de Floridablanca, Lebrija, Piedecuesta, Girón, Bucaramanga, Tona, Charta y Suratá en Santander, se construyó una escuela agroecológica cuyo objetivo es la organización campesina para aprender del otro, reflexionar sobre los recursos de la zona y realizar trueques en los mercados campesinos ^28^.

El fortalecimiento del campesinado, surgido como una apuesta de los gobiernos locales y las organizaciones sociales de base, ha dado frutos en la cohesión del tejido social de los territorios. Así lo demuestra el impulso de la competitividad de los campesinos a partir de prácticas sostenibles y agroecológicas, cada vez más en auge entre los productores del campo. Esto, además, potencia sus conocimientos tradicionales y ayuda a la preservación de la naturaleza ^29^.

Todos los colombianos habrían podido alimentarse durante la cuarentena gracias al trabajo de los campesinos del país, pero, a pesar de que ellos han sido un bastión para garantizar la seguridad alimentaria del país, la pandemia ha implicado enfrentarse a retos de transporte, adquisición de insumos, mano de obra, variabilidad e incertidumbre en los precios, falta de créditos, sobreproducción y disminución de la demanda de algunos alimentos. Según la Universidad El Bosque, el 87 % de los habitantes del campo dedicados a actividades agro-pastoriles ha enfrentado dificultades económicas a consecuencia de la pandemia ^30^. Por ello, las estrategias que buscan su beneficio en un país con vocación agrícola siempre contribuirán al bienestar y calidad de vida de toda la población. Se ha planteado que la afectación del sector agrícola en términos productivos y económicos hubiera sido nula durante la cuarentena ^31^ de no haber sido porque la importación de alimentos no cesó ^32^.

La pandemia del coronavirus ha dejado una lección importante en torno a las estrategias que contribuyen a la seguridad y soberanía alimentaria. En este sentido, el trueque y el fortalecimiento de los sistemas alimentarios locales mediante el impulso a los mercados campesinos, se convierten en mecanismos potencialmente exitosos centrados en la alimentación desde y para los pueblos.
